# Motor cortical regulation of sparse synergies provides a framework for the flexible control of precision walking

**DOI:** 10.3389/fncom.2013.00083

**Published:** 2013-07-11

**Authors:** Nedialko Krouchev, Trevor Drew

**Affiliations:** Groupe de Recherche sur le Système Nerveux Central, Département de Physiologie, Université de MontréalMontréal, QC, Canada

**Keywords:** locomotion, motor cortex, voluntary gait modifications, cat, synergy

## Abstract

We have previously described a modular organization of the locomotor step cycle in the cat in which a number of sparse synergies are activated sequentially during the swing phase of the step cycle (Krouchev et al., 2006). Here, we address how these synergies are modified during voluntary gait modifications. Data were analysed from 27 bursts of muscle activity (recorded from 18 muscles) recorded in the forelimb of the cat during locomotion. These were grouped into 10 clusters, or synergies, during unobstructed locomotion. Each synergy was comprised of only a small number of muscles bursts (sparse synergies), some of which included both proximal and distal muscles. Eight (8/10) of these synergies were active during the swing phase of locomotion. Synergies observed during the gait modifications were very similar to those observed during unobstructed locomotion. Constraining these synergies to be identical in both the lead (first forelimb to pass over the obstacle) and the trail (second limb) conditions allowed us to compare the changes in phase and magnitude of the synergies required to modify gait. In the lead condition, changes were observed particularly in those synergies responsible for transport of the limb and preparation for landing. During the trail condition, changes were particularly evident in those synergies responsible for lifting the limb from the ground at the onset of the swing phase. These changes in phase and magnitude were adapted to the size and shape of the obstacle over which the cat stepped. These results demonstrate that by modifying the phase and magnitude of a finite number of muscle synergies, each comprised of a small number of simultaneously active muscles, descending control signals could produce very specific modifications in limb trajectory during locomotion. We discuss the possibility that these changes in phase and magnitude could be produced by changes in the activity of neurones in the motor cortex.

## Introduction

The question of modularity within the locomotor control system has a long history. From the original proposition of Graham Brown (1911, 1914) that two half-centres are responsible for generating locomotion grew a body of experimental work to determine the neuronal basis of the alternating rhythmical activity observed during locomotion (Jankowska et al., 1967a,b; Lundberg, 1981). This work took a new and, initially, controversial, direction when Grillner postulated the existence of a central pattern generator (CPG) for locomotion (Grillner and Zangger, 1975, 1979; Grillner, 1981). Subsequent studies have shown that this CPG in the spinal cord has the intrinsic ability to generate an intricate and complex pattern of locomotor activity (Pearson and Rossignol, 1991). From the original concept of the CPG as a single neuronal entity (or network) has grown the idea that the CPG may in fact be considered as a series of modules that are interconnected and provide the capacity to produce a rich behavioral repertoire, involving flexible and coordinated activity around multiple joints.

A key evolution in this respect was the formulation of the concept that the CPG is comprised of a number of unit pattern generators (Grillner, 1981; Grillner and Wallen, 1985). Grillner suggested the existence of four pairs of unit pattern generators responsible for producing the rhythmical pattern of activity in the hip, knee, ankle, and toe muscles. He proposed that relatively simple command signals to modify the connections between the hip and knee modules, for example, could easily change the pattern of muscle activity required for forward progression into that required for walking backwards.

In the turtle spinal cord, such an organization of interconnected modules has been demonstrated electrophysiologically to generate several different scratch patterns depending on the location of the offending stimulus (Berkowitz and Stein, 1994; Stein and Smith, 1997). Additional evidence for modularity in the turtle has come from the studies of deletions, in which bursts of activity in some populations of interneurones and their associated motor pools are absent in some scratch cycles while others persist (Stein and Daniels-McQueen, 2002; Stein, 2008). However, the neuronal mechanisms leading to modularity within the mammalian spinal cord are more difficult to study because of the size and complexity of the neuronal networks. As in the turtle, some evidence for modularity, although not necessarily for independent burst generators, has also come from the study of deletions (Grillner and Zangger, 1979; Jordan, 1991; Smith et al., 1995; Lafreniere-Roula and McCrea, 2005; Zhong et al., 2012). These deletions are observed in flexor and extensor motoneurones and, in many cases, have no effect on cycle timing, supporting views that rhythm generation (defining the basic rhythmicity of the step cycle) and pattern generation (defining the spatio-temporal organization of the muscle bursts within the step cycle) are separate (Lennard, 1985; Koshland and Smith, 1989). These results have led to a particularly interesting computational model of locomotion consisting of a rhythm generator and distinct pattern generators (Rybak et al., 2006; McCrea and Rybak, 2007; Zhong et al., 2012).

As an extension of these ideas, and particularly on the basis of Grillner's unit CPG model, we suggested (Drew, 1991a) that the existence of such modules could provide a substrate by which the motor cortex could exert a precise control over the magnitude, duration and relative timing of specific muscles groups while at the same time ensuring that these changes are appropriately integrated into the locomotor cycle. Similar ideas were incorporated into computer simulations designed to determine how supraspinal command signals might interact with spinal unit CPGs in a human model of locomotion (Taga, 1995, 1998).

More recently, the idea of modularity within the spinal circuits has been developed to incorporate the idea of muscle synergies. This development, which owes much to the studies of Bizzi and his collaborators (Bizzi et al., 1991; Tresch et al., 1999, 2002; d'Avella et al., 2003; d'Avella and Bizzi, 2005), posits that the nervous system produces complex movements by combining the activity of a limited number of synergies, 4–6 in most studies. In brief, synergies are defined mathematically, by using decomposition methods such as non-negative matrix factorization (NNMF), as a matrix of weights that differentially activate *all* of the muscles involved in producing a movement. By modifying the magnitude and the phase of activity of each synergy, a wide range of movement patterns can be produced (Tresch and Jarc, 2009). Such synergies have been described during locomotion, scratching and swimming in the frog (Giszter et al., 1993; Saltiel et al., 2001; Cheung et al., 2005), during postural compensation to perturbation in cats and humans (Ting and Macpherson, 2005), during human locomotion (Ivanenko et al., 2004; Lacquaniti et al., 2012) as well as in reaching movements in primates and humans (d'Avella et al., 2006, 2008; Overduin et al., 2008). Muscle synergy analysis has also been used to study the deficits in movement after stroke (Cheung et al., 2009, 2012; Clark et al., 2010). In some of these experiments, and particularly those performed in spinal animals, these synergies have been suggested to form the basis of unit burst generators of the type proposed by Grillner (Hart and Giszter, 2004; Cheung et al., 2005).

In our own studies of synergies (Krouchev et al., 2006; Drew et al., 2008a), we have taken a different approach in which synergies are defined using more classical, physiological methods. In our approach, a synergy is defined as a group of muscles that are synchronously activated such that the period of activity during locomotion begins and ends simultaneously in all muscles in a synergy. By applying a custom clustering algorithm we were able to identify 11 synergies in the forelimb of the cat during unobstructed locomotion, with nine of these occurring during the swing phase. These synergies differed from those identified most commonly by using mathematical decomposition methods (see above) in that they were both more numerous and contained only a small proportion of the total number of muscle bursts recorded. We refer to these as sparse synergies to differentiate them from those synergies comprised of all periods of muscle activity (see preceding paragraph). Our suggestion, as in our original work on this issue (Drew, 1991a), is that the motor cortex modulates these sparse synergies in order to modify limb trajectory during locomotion (Drew et al., 2008a,b).

The question remains, however, as to *how* these synergies are modified during voluntary gait modifications and whether the modifications in synergies that are observed are compatible with the discharge activity of neurones in the motor cortex recorded during the same behaviors. To answer that question, we have extended our previous analysis to examine how synergies are modified when cats step over obstacles. We consider two conditions. First we examine how synergies are modified in the lead (when a given limb is the first to step over an obstacle) and the trail (second to step over the obstacle) condition, for which our previous studies showed differential patterns of muscle activation related to the biomechanical requirements of the gait modification (Drew, 1993; Lavoie et al., 1995; McFadyen et al., 1999). Second, we examine how the synergies are modified when the cats step over obstacles of different shapes and sizes (Drew, 1988, 1991b, 1993). We then discuss the implications of these results for the cortical control of voluntary gait modifications.

## Methods

The methods used in this study were either identical or similar to those previously detailed (Krouchev et al., 2006) and will only be briefly described here. All animals used in this study were originally used for other studies (Drew, 1993; Stapley and Drew, 2009; Yakovenko et al., 2011). Details concerning the methods used for animal training and implantation methods in the different animals used in the current manuscript can be found in those manuscripts. Details concerning the analytical methods are found in Krouchev et al. (2006).

### Training

Cats were trained to walk on a treadmill at 0.35–0.5 m.s^−1^, first in the absence of any obstacles (unobstructed locomotion) and then in the presence of 1 or 2 obstacles attached to the treadmill belt. In these experiments, the obstacles always moved at the same speed as the treadmill. The obstacles were visible to the cat a minimum of two steps before the step over the obstacle. Most data were obtained from studies in which the cat stepped over a cylindrical obstacle of 10 cm cross-section. Additional data were obtained during steps over a high obstacle (13 cm high, 1 cm wide), a small high obstacle (7.5 cm high, 1 cm wide) and a wide obstacle (2.5 cm high, 15 cm wide) (see Drew, 1993 and Figure 8E).

### Surgery

After the cats were trained, they were prepared for surgery under general anesthesia and in aseptic conditions following the protocols approved by the animal ethics committee at the Université de Montréal and according to the recommendations of the Canadian Council for the Protection of Animals. Details for different animals can be found in the references above. Heart rate and temperature were monitored during surgery and anesthesia level was regularly verified by testing for a corneal reflex. Solutes were administered throughout the surgery and analgesics were provided prior and subsequent to the surgery.

In all animals, pairs of Teflon-insulated, braided stainless steel wires were implanted into multiple muscles of the left forelimb. Wires were led sub-cutaneously to a 51 pin connector attached to the cranium of the cat. Although other surgical procedures were practiced on these cats, they are not reported here, as they are not relevant to the data illustrated. Details of these supplementary procedures can be found in the published manuscripts (see above). All data presented in this manuscript were obtained several weeks following the surgical procedures.

### Protocol

Electromyographic (EMG) activity was normally recorded along with cell activity during locomotion. Data were generally recorded during unobstructed locomotion and then during periods of locomotion when cats stepped over 1 or 2 obstacles attached to a treadmill belt. EMG data were filtered at 100 Hz to either 450 or 500 Hz and amplified by a factor of 1–10 K to produce a signal of ~1 volt. Data were digitized at 1 KHz. Video recordings of the locomotion were obtained simultaneously with the EMG data and synchronized with the aid of a SMPTE (Society of motion pictures and television engineers) digital time code.

### Analysis

**Figure 1:**
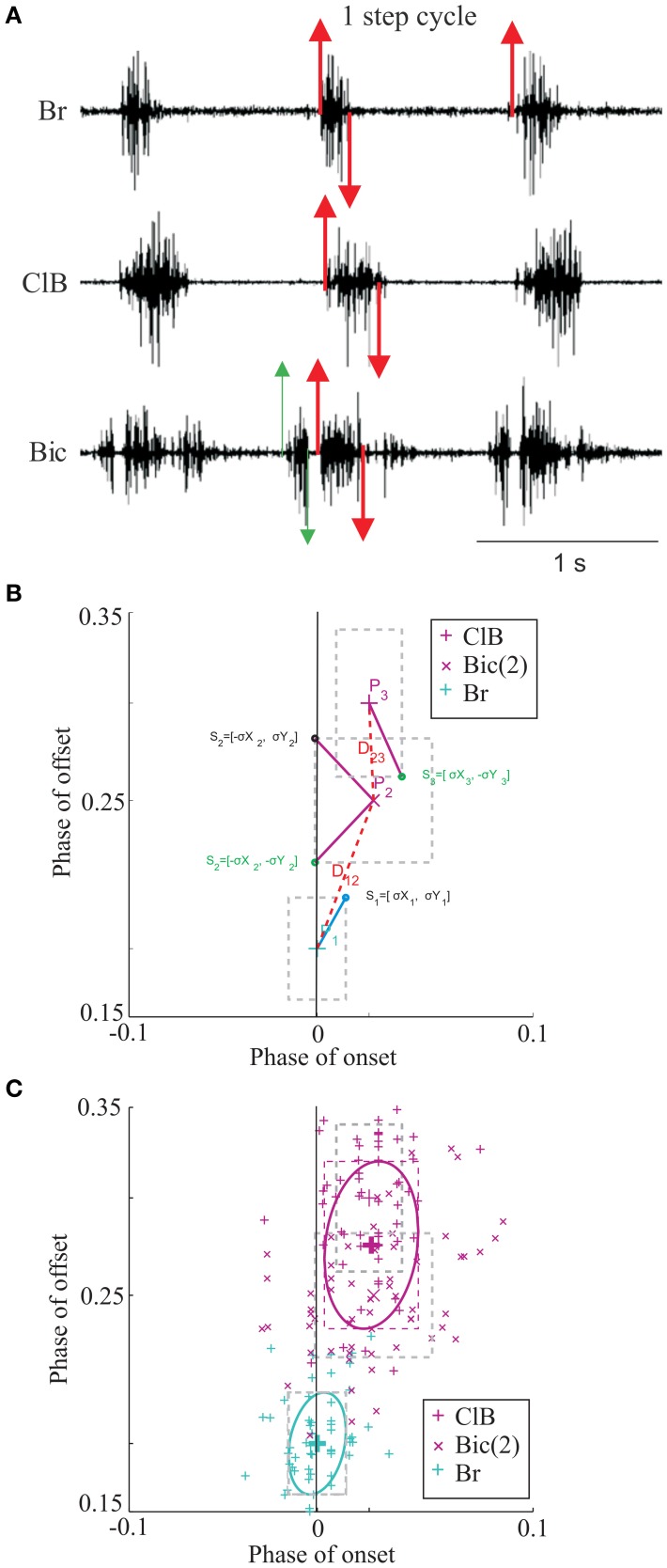
**Data selection and clustering algorithm. (A)** Untreated data during locomotion from the brachialis (Br), Cleidobrachialis (ClB), and Biceps brachii (Bic) muscles. Upward and downward directed arrows indicate onset and offset of muscle burst activity, respectively. All activity was synchronized to the Br and burst onset activity is defined as phase = 0.0. A step cycle is defined as the time between two successive bursts (phase = 1.0). Activity in other bursts is defined with respect to Br onset. The red arrows indicate data illustrated in **(B,C)**. The green arrows define the first burst of activity in Bic [Bic(1) in Figure 2]. **(B)** The rectangles define 1SD of the mean onset and offset of the activity of each muscle (P1–P3, individual data illustrated in **C)**. A vector is drawn from the center of each rectangle to the vertex closest to the center of the nearest neighbor (thick lines, Sn). The vector distance (dotted line: Dn) is then calculated as described in the text. **(C)** Bivariate ellipses are drawn around the centroid of each cluster (see text). Same dataset as in Figure 2.

**Figure 2:**
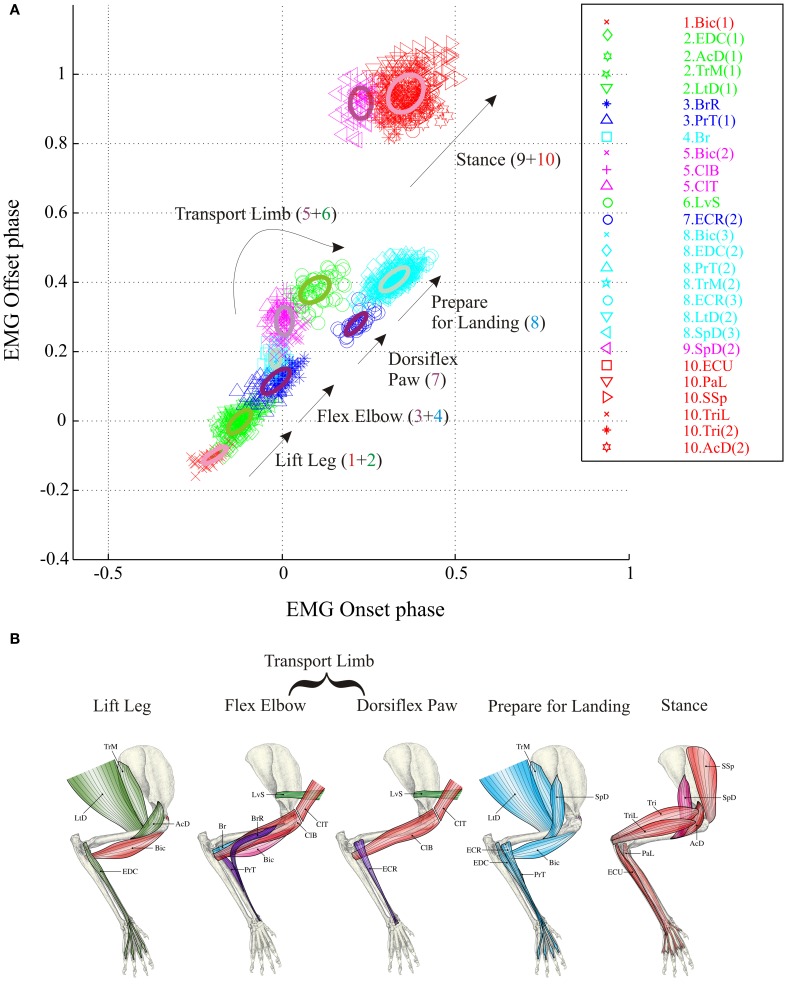
**Synergies during unobstructed locomotion. (A)** The results of the cluster analysis are illustrated for a total of 27 periods of EMG activity recorded from 18 muscles in 3 cats (30–70 values for each muscle burst). 10 clusters, each corresponding to a muscle synergy, are identified by color and the EMG bursts in each cluster are identified by symbol and color as shown in the key to the right. The same order of muscles is used in the key of all figures except when a change in synergy composition makes it impossible. The spatial location of each cluster is illustrated by the ellipses (thick lines). EMG onsets and offsets are referenced to the onset of activity in the brachialis (Br) muscle (phase = 0.0). Negative phase onsets indicate muscle bursts active prior to the onset of activity in the Br. **(B)** the muscles used in this study are illustrated on stick figures of the cat's forelimb taken from Crouch (1969). Clusters are organized according to the biomechanical function and the colors of the muscles correspond to the colors of the synergies. Note that colors accorded to some muscles are different from those used in Krouchev et al., 2006 because of a slightly different composition of the synergies (see text). Muscle Abbreviations: AcD, acromiodeltoideus; BrR, brachioradialis; ClT, cleidotrapezius; ECR, extensor carpi radialis; EDC, extensor digitorum communis; ECU, extensor carpi ulnaris; LtD, latissimus dorsi; LvS, levator scapularis; PaL, palmaris longus; PrT, pronator teres; SpD, spinodeltoideus; SSp, supraspinatus; Tri, triceps bacchii, long head; TriL, triceps brachii, lateral head; TrM, teres major.

**Figure 3:**
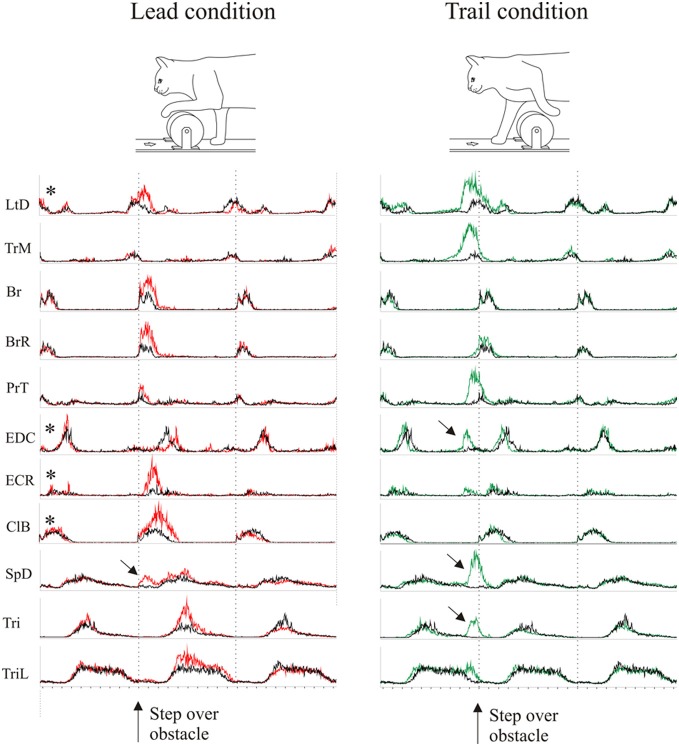
**Averaged EMG activity during voluntary gait modifications**. The figure shows the activity of 11 representative muscles during the lead (left column, red traces) and the trail (right column, green traces) condition. Average activity during gait modification is superimposed onto the activity in the unobstructed condition (black traces). Data are synchronized to the onset of the Br and the duration is normalized to the average control step cycle duration. The amplitude of the activity in each EMG is scaled to the same value for all three traces (lead, trail and unobstructed). EMG traces with an asterisk are taken from cat MC29, those without an asterisk are from cat RS26. Arrows indicate bursts of activity that were not present in the unobstructed condition.

**Figure 4:**
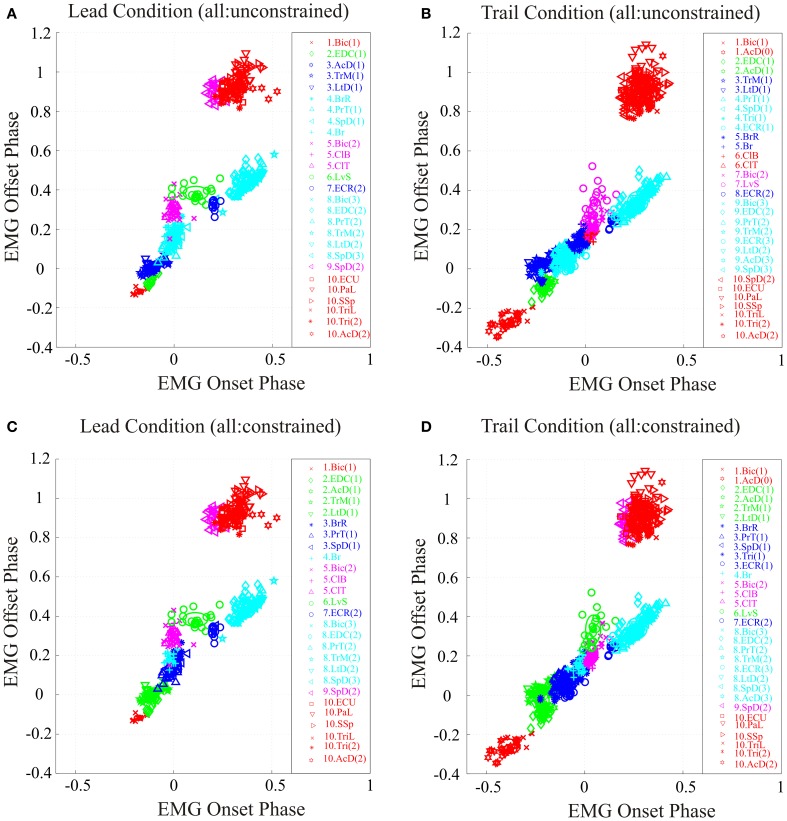
**Synergies during the lead and trail conditions. (A,B)** clusters formed by applying the analysis to all bursts recorded in each condition and allowing the analysis to define the resulting clusters (unconstrained). **(C,D)** analysis performed using all bursts but with the clusters constrained to have the identical composition as in the unobstructed condition (Figure 2). Note that in the unconstrained condition, the colors describing some clusters are different from those used in Figure 2 because of the modification of synergies detailed in the text. In the constrained condition, the colors are identical to those used in Figure 2.

**Figure 5:**
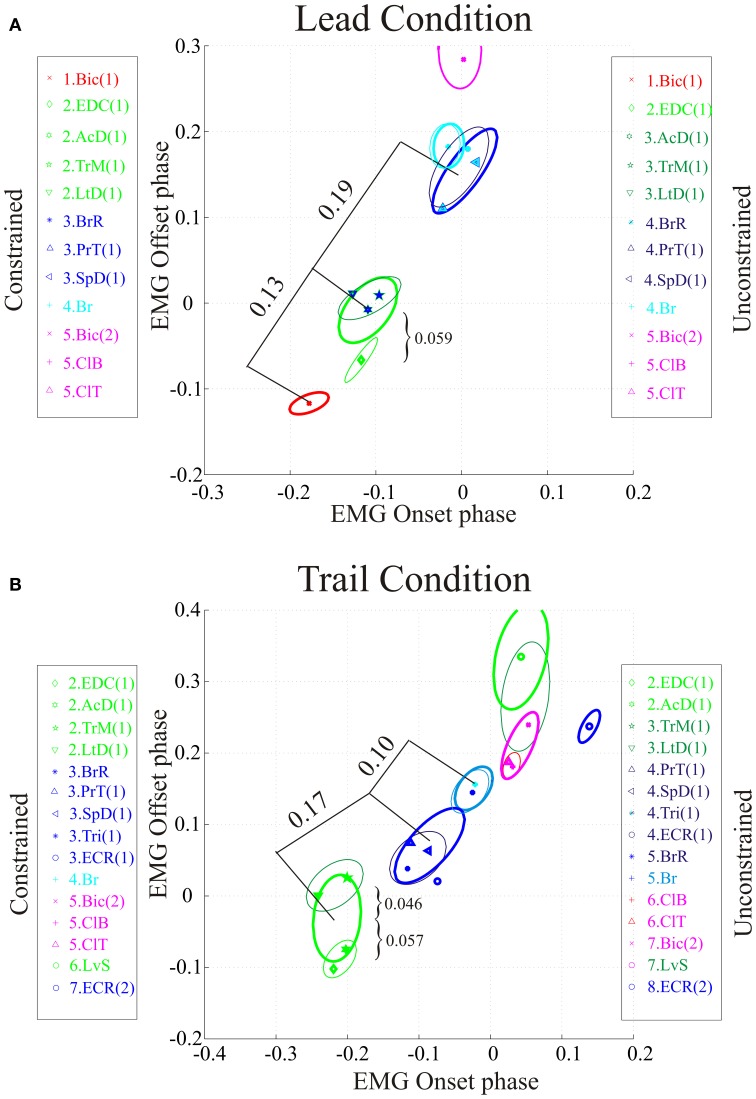
**Comparison of synergies in the constrained and unconstrained conditions**. Data are illustrated for the first 5 clusters in the lead condition **(A)** and for clusters 2–8 in the trail condition **(B)**. Ellipses indicating clusters calculated using the constrained condition are illustrated as thick lines and those from the unconstrained condition as thin lines. Each symbol represents the centroid of a burst of activity in a given muscle as indicated by the key for the constrained condition (to the left of each illustration). It should be emphasized that the location of the centroid of each muscle is identical for the constrained and unconstrained condition. Numerical values on the plots indicate the Euclidean distance between the centroids of adjacent constrained clusters (straight lines) or, in **(B)**, between the centroids of the constrained and unconstrained clusters ({). Cluster #1 in the trail condition is not displayed because of the scale used. Note that the colors identifying each muscle and each ellipse are the same for the constrained and unconstrained conditions and identical to those used in Figure 2. As a result, muscles with the same colors sometimes belong to different clusters in the unconstrained condition (see key).

**Figure 6:**
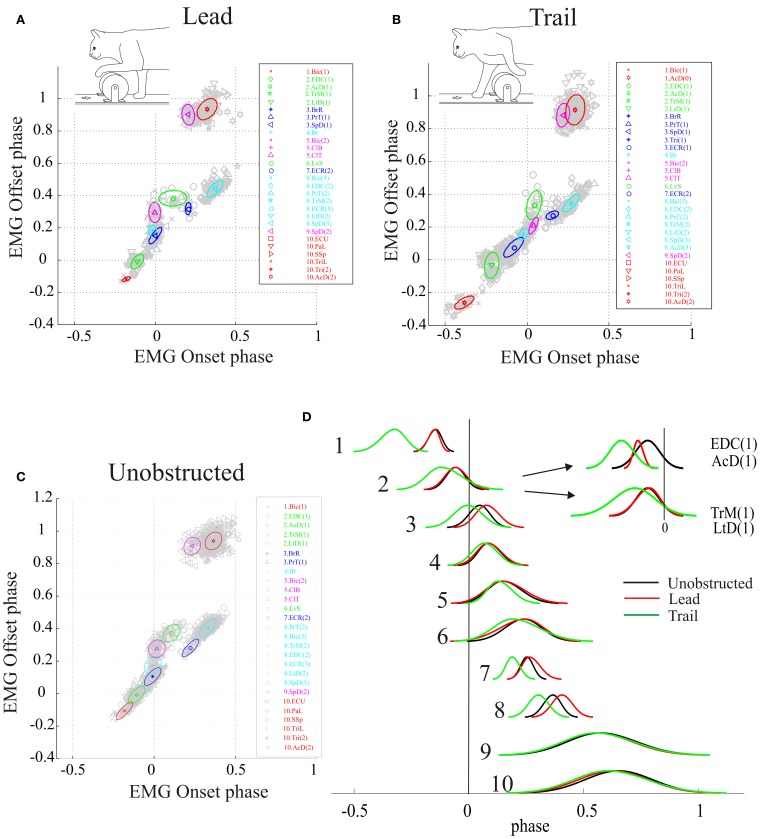
**Changes in phase of activity of synergies during gait modifications. (A–C)** centroids of the synergies obtained in the lead **(A)**, trail **(B)** and unobstructed **(C)** conditions. **(D)** direct components showing the changes in phase of the synergies in the lead (red traces) and trail (green traces) compared to the unobstructed condition (black traces). The inset in **(D)** divides synergy #2 into two synergies, as suggested by the analysis in the unconstrained condition (Figures 4, 5).

**Figure 7:**
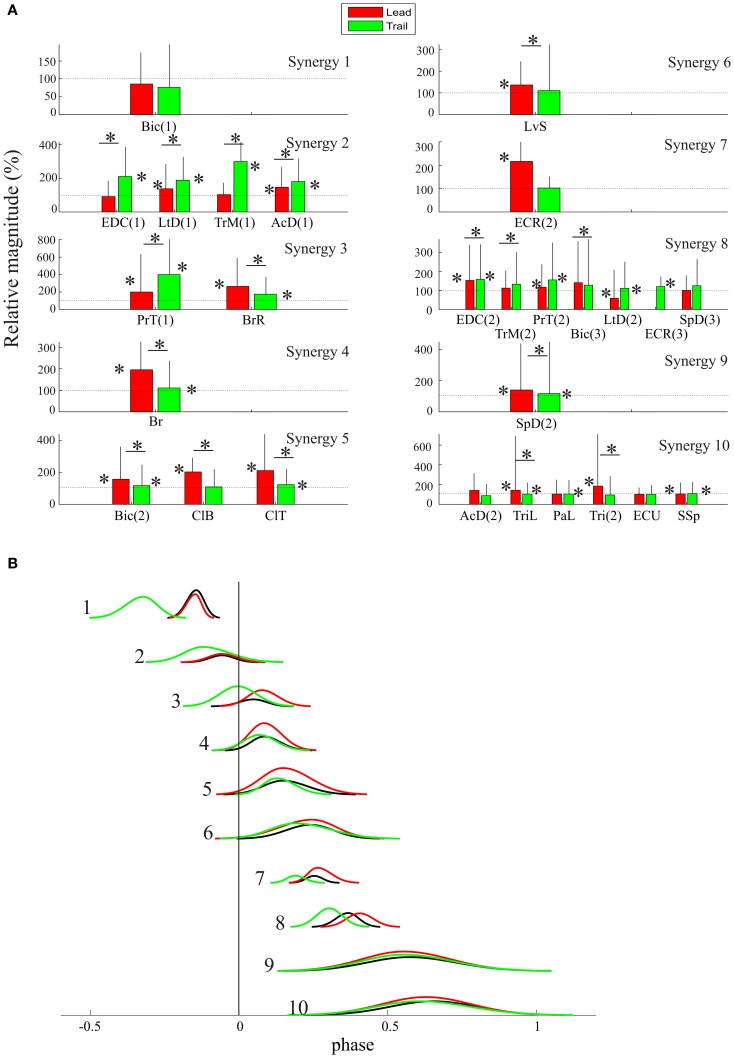
**Change in magnitude of the synergies. (A)** the mean magnitude (+SD) of the activity in each muscle during lead (red bars) and trail (green bars) is expressed as a percentage of the activity in the unobstructed condition (100%, thin horizontal line). The magnitude is normalized to the duration of the burst (see text). Asterisks beside the bars indicate muscle bursts that were significantly different from control (*p* < 0.05). Horizontal lines with an asterisk indicate muscle bursts that were significantly different between the lead and trail conditions. **(B)** the average change in the level of activity from each of the synergies is used to modify the magnitude of the direct components, which now provide a representation of both the change in phase and the change in magnitude of the synergies that is required to step over the obstacles in the lead and trail condition.

**Figure 8:**
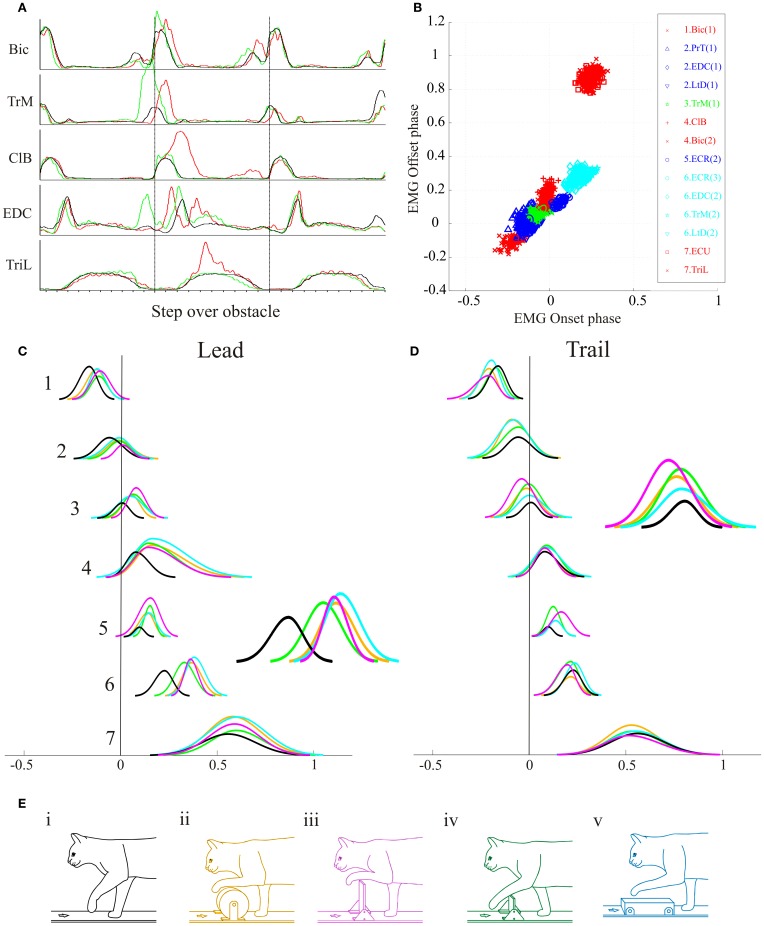
**Modification of synergies during steps over obstacles of different size and shape. (A)** averaged activity of 5 representative muscles during the lead (red), trail (green) and unobstructed (black) conditions. Data are synchronized to the onset of activity in the ClB. **(B)** Cluster analysis for the database of 14 muscle bursts available from this cat. **(C,D)** direct component analysis illustrating the relative changes in phase and magnitude of each synergy when the cat steps over obstacles of different sizes and shapes in the lead **(C)** and trail **(D)** conditions. The insets amplify the traces representing synergy #6 in the lead condition and synergy #3 in the trail condition. The color code for the obstacles is represented by the color of the cats in **(E)**.

The video recordings were initially screened for sections of stable locomotion in which the cat maintained its position in the center of the treadmill. As such, we chose sections of data in which EMG activity was uniform from cycle to cycle with no evidence of intermittent changes. We then manually marked the onset and offset of each burst of EMG activity within these sections (Figure 1A) and classified them as either steps over the obstacle with the left leg leading or trailing, or as unobstructed locomotion (either no obstacles attached to the treadmill or at least two steps before a step over an obstacle; see Drew, 1993). A step cycle was defined as the time between two successive periods of activity in either the brachialis (Br; Figures 1–7) or the cleidobrachialis (ClB; Figure 8) muscle, each of which becomes active at approximately the onset of swing. The step cycle was normalized to unity (1.0) and the onsets and offsets of periods of activity in each selected muscle were then expressed as a proportion, or phase, of the step cycle. Measured events occurring after the onset of the ClB or Br were given positive values while those occurring before ClB or Br onset were given negative values. Data were plotted in phase space in which the phase of offset of a given burst was plotted as a function of its onset (Figures 1C, 2A).

#### Associative clustering

Trial data points are formed pairwise in the phase plane (*x* = *onset, y* = offset) and one or more bursts may be *associated* in clusters as described in Krouchev et al. (2006). It is important in this respect to realize that the data points for each muscle are defined as belonging to that muscle and cannot be divided among different clusters. In the clustering algorithm, it is assumed that each burst is fully described by its centroid—i.e. the mean (onset, offset) phase vector, and the associated pair of standard deviations (SD's: σ*X*, σ*Y*; assumed uncorrelated). Hence, each burst is assumed equivalent to the encompassing rectangle ***[***−σ*X*, σ*X****]*** × ***[***−σ*Y*, σ*Y****]*** around the centroid mean values ***[****X, Y****]*** (Figure 1B).

Sufficiently overlapping rectangles form clusters, or synergies (Krouchev et al., 2006). Thus the algorithm associates sets of data points from different muscles rather than dissociating them as do most clustering methods.

First, the individual data points are tested for outliers using Rosner's test (Rosner, 1983) with a 2 SD margin. For each burst ***i***, the phase-plane vectors of mean phase:

$$P_{i}=\left(X_{i},Y_{i}\right)=mean\left(onset_{i},offset_{i}\right)$$

and of standard deviations

$$S_{i}=\left(\sigma X_{i},\sigma Y_{i}\right)=std\left(onset_{i},offset_{i}\right)$$

are calculated. The distance between two bursts ***i*** and ***j***, is expressed as the phase-plane vector (Figure 1B):

$$D_{ij}=P_{i}-P_{j}$$

Two bursts are considered part of the same cluster when:

(1) $$q_{ij}=max\left\{\left(P_{i}\pm S_{i}\right)D_{ij}+\left(P_{j}\pm S_{j}\right)D_{ij}-∥D_{ij}{∥}^{2}\right\}/∥D_{ij}∥>0$$

where the maximum is taken over all possible combinations (Figure 1B), the products are the dot (scalar) vector products, and ∥·∥ is the usual Euclidean norm in the xOy phase plane.

For all possible pairs of bursts ***i*** and ***j*** (*i* = 1,2 …, *n, j* = *i* + 1, …, *n*)— where ***n*** is the total number of bursts to classify, Equation (1) is verified and thence a boolean ***n*** × ***n*** upper-triangular adjacency matrix **B** is obtained (**B**_*ij*_ = 1 when **q**_*ij*_> **0**, and zero otherwise). The latter adjacency matrix is then the input to a Matlab implementation of an algorithm due to Press et al. (1992) to determine the equivalence classes—i.e., the clusters. The final cluster numbers are rearranged so that their mean onsets proceed in an ascending order.

The clustering proceeds in two sub-stages. First, we cluster the bursts coming from the first (and most elaborate and ample) subset of the data—coming from the same animal. In the second sub-stage, additional bursts (coming from additional animal data sets) are either allowed to join an existing cluster, or to form a new one. In cases in which the existing cluster has more than one member, the new candidate burst will join if it overlaps with at least two bursts from the existing cluster. It should be noted that this associative clustering method is robust to even relatively large changes in the centroids of EMGs making up a cluster. In a previous publication (Krouchev et al., 2006; supplemental information), we tested the stability of the methods by introducing random jitter to the centroids of the muscles making up a cluster. In 970/1000 cases in which each centroid was displaced by <0.6 SD, there was no change in cluster composition compared to that obtained using the actual data.

### Constrained vs. unconstrained clusters

In the analysis in our original papers (Krouchev et al., 2006; Drew et al., 2008a) the algorithm was always unconstrained in that muscles were formed into clusters, and therefore synergies, simply on the basis of the rules summarized in the preceding paragraph. A similar approach was used to obtain the synergies active during unobstructed locomotion (Figure 2) as well as during the lead and trail conditions of the voluntary gait modifications. However, because of the addition of periods of muscle activity that occurred only during the gait modifications, and because of the relative changes in phase of some muscles, there were small changes in the composition of some of the synergies. This makes a direct comparison of changes in the phase and magnitude of synergies during all three conditions problematic. We, therefore, also used a constrained analysis in which muscles bursts were confined to the same clusters identified during unobstructed locomotion. This allowed us to directly compare the changes in phase and magnitude of synergies comprised of the same muscles in all three conditions. Further details of the approach and of its limitations are provided in the Results and Discussion.

#### Direct component analysis (DCA)

For each cluster we derive a temporal activation profile, which is labeled a *direct* component (DC). The latter contrasts to the muscle activation components in the literature, which most often are obtained through abstract mathematical decomposition.

DCs are derived directly from the corresponding overall **(**onset, offset**)** phase statistics, which describe the periods of activity of the muscles (or bursts) forming the cluster. For each cluster, we calculate the 2 *marginal univariate* Gaussian probability density functions (pdf) for the onset and the offset phases. For each cluster ***k***, the onset/offset pdf is, respectively, *N*(*X*_*k*_, σ*X*_*k*_) and *N*(*Y*_*k*_, σ*Y*_*k*_) and activation of the muscles forming this cluster is assumed to span the phase interval (*X*_*k*_ − 3σ*X*_*k*_, *Y*_*k*_ + 3σ*Y*_*k*_).

We further assume that the overall shape of the temporal activation profile for cluster ***k*** is captured by Gaussian basis-functions. Hence its DC ***u***_***k***_***(t)*** is described by:

$$u_{k}\left(t\right)=\{\begin{array}{l} \exp\left(-{\left(t-Z_{k}\right)}^{2}/2\sigma_{1}\right),\;t\;<Z_{k}\\ \exp\left(-{\left(t-Z_{k}\right)}^{2}/2\sigma_{2}\right),\;t\;>Z_{k} \end{array}$$

where

$$\begin{array}{l} Z_{k}=(X_{k}+Y_{k})/2,\sigma_{1}=(Z_{k}-X_{k})/3+\sigma X_{k},\text{ and}\\ \sigma_{2}=(Y_{k}-\;Z_{k})/3\;+\sigma Y_{k} \end{array}$$

#### Cluster statistics and representation

In this paper we use a correlated bivariate-normal model. The data scatter for burst ***k*** is captured by the pdf:

(2) f(x,y) ~ exp(−z/2w)

where:

$$\begin{array}{l} z=u^{2}+v^{2}-2\;uv\rho \;w\;=\;1\;-\rho^{2}\\ u=\left(x-X_{k}\right)/\sigma X_{k}\;v\;=\left(y-Y_{k}\right)/\sigma Y_{k} \end{array}$$

Hence, each cluster is represented by an ellipse of the form:

(3) $$u^{2}+v^{2}-2\;uv\rho =1-\rho^{2}$$

The ellipses' skewing and orientation depend on the value of **ρ**. Krouchev et al. (2006) assumed no (x,y) correlation—i.e., **ρ** = 0. This yielded ellipses with major axes, respectively (σ*X*, σ*Y*), parallel to the coordinate axes. For the general case of non-zero **ρ**, it may be shown that the ellipse described by Equation (3) touches the ±1*SD* encompassing rectangle exactly at the points (±**1**, ±**ρ**) and (±**ρ**, ±**1**) (Figure 1C).

#### Statistics

To determine whether the phase and/or the magnitude of the synergies were significantly different in each condition we used ANOVAs across the conditions (unobstructed, lead and trail). Individual One-Way ANOVA's were performed for each of the synergies using the data for the individual periods of activity of each muscle within a synergy. Tests for difference in the phase of activity were made using the onset ~ *N*(*X*_*k*_, σ*X*_*k*_), the offset ~*N*(*Y*_*k*_, σ*Y*_*k*_) and the peak phase (*X*_*k*_ + *Y*_*k*_)/2 of activity. The null-hypothesis for each ANOVA is that condition has no significant effect on the range of phase-values in the sample. For synergies that showed a significant (*p* < 0.05) effect of the locomotion condition, we performed pair-wise *t*-tests between conditions. Individual One-Way ANOVA's were also performed to determine if the magnitude of the synergies also varied as a function of condition (see Results).

## Results

### Database

The database for the principal analysis in this paper is based around cat RS26, as in a previous publication (Krouchev et al., 2006) examining synergies during unobstructed locomotion. Supplementary muscles in that previous publication were obtained from 3 other cats, PCM3, MC8 and RS23. Ideally, we would have used the same database in the current manuscript. Unfortunately, however, data for voluntary gait modifications were not available from all animals. We have, therefore, complemented the data from RS26 with data from cats MC29 and RS23 for the current analysis to produce a full dataset during unobstructed locomotion consisting of 27 bursts of EMG activity from 18 muscles of the forelimb, all recorded on the same side. The 10 clusters defining the muscle synergies in this dataset during unobstructed control locomotion are illustrated in Figure 2 and the average activity of some of these muscles can be observed in Figure 3 (black lines). Note that the clusters defined in the present analysis are very similar to those published previously (Figure 6 in Krouchev et al., 2006). The major difference is that the current dataset produced 10 clusters of activity rather than the 11 that we defined previously. This was primarily caused by the muscles in cluster #2 forming a single cluster (including EDC, LtD and TrM) rather than being divided into 2 clusters as in our previous analysis. One other difference is the presence of a very early burst of activity in the Bic, preceding activity in all other muscles. Apart from these minor differences with our previously described dataset (Krouchev et al., 2006), the essential features of these synergies are maintained. These include: the sequential pattern of activation of the synergies; the sparse nature of each synergy; the fact that a given muscle may be represented in more than one synergy and the fact that a given synergy can include muscles acting proximally together with others acting distally (e.g., EDC and TrM in cluster #2).

### Changes in phase of synergies during gait modifications (lead and trail conditions)

During the gait modifications, there were substantial changes in the magnitude and phase of some of the bursts of EMG activity as we have also described elsewhere (Drew, 1993; Lavoie et al., 1995; Drew et al., 2008a). The changes observed in selected muscles of the current dataset are illustrated in Figure 3 for the lead and trail condition. The EMG data for all muscles are normalized in time to the average cycle duration for the original dataset in which they were recorded, and normalized in amplitude to the largest magnitude recorded from any one given muscle. As such, changes in amplitude and relative changes in phase are clearly visible in the presentation. Several points need to be emphasized. First, in both conditions, there are changes in amplitude and phase in a number of muscles. During the lead condition, these changes are primarily expressed as changes in amplitude (e.g., Br and BrR) with fewer changes in phase, although see phase delay in EDC. In contrast, during the trail condition, there are major changes in both phase and amplitude in a number of muscles. This is particularly clear in the shoulder retractor muscles (LtD and TrM) and in the muscles acting around the wrist and digits (PrT, EDC). Last, one other major difference from the control activity is the presence of periods of activity during the gait modifications (Figure 3, arrows) that were not present in the unobstructed condition. The clearest examples are the supplementary periods of activity in the EDC, SpD and the Tri at the onset of swing in the trail condition. There are also small changes in some of the extensor muscles. These occur after the step over the obstacle in the lead condition and before the step over the obstacle in the trail phase.

In general, most of the changes in phase of the muscles active during swing occur during the trail condition, before the onset of swing (defined here as Br onset) while the more modest phase changes in lead occur after swing onset. As discussed previously (Drew, 1993; Lavoie et al., 1995; McFadyen et al., 1999), this difference is related to the constraints of the task. During the lead condition, swing begins with the obstacle well forward of the paw and the major requirement is to lift the limb above and over the advancing obstacle. During the trail condition, swing begins with the obstacle close to the trail paw and the major requirement is to retract the limb sufficiently to ensure that the limb is lifted away and above the obstacle as it continues to advance (see Drew, 1993).

To determine the effects of the changes in the pattern of muscle activity on synergy composition, we performed the cluster analysis on the datasets for the voluntary gait modifications (lead and trail conditions) using all available bursts of EMG activity including those that were present only during the voluntary gait modifications (Figure 4). As explained in the Methods, we used two complementary approaches to examine changes in synergy. In the first instance, we applied the clustering algorithm to the entire dataset in the lead and the trail condition in exactly the same way as we did for the unobstructed data. We refer to this as the unconstrained condition (Figures 4A,B). In this approach, we are effectively asking whether the same synergies are found in all three conditions studied, unobstructed, lead and trail. In the second approach, and the one that we used for all further analyses, we constrained the synergy composition during the lead and trail conditions to be identical to that observed in the unobstructed condition (Figures 4C,D). In this instance, we are determining how the phase of the synergies identified during unobstructed locomotion needs to be modified in order to obtain the patterns of activity observed during the gait modifications. In this second approach, we used the adjacency matrix to determine to which cluster the bursts activated only during the gait modifications were associated. This, constrained, approach has the advantage of allowing us to provide a direct comparison of the changes in phase and magnitude that occurred in each synergy during these gait modifications.

The results obtained by using the unconstrained algorithm show that the muscles bursts observed during the voluntary gait modifications are organized into synergies that are fully consistent with those observed in the unobstructed condition. Specifically, the analysis results in the presence of a similar number of synergies as in the unobstructed condition, with the same sequential organization as in unobstructed locomotion and with each synergy being comprised of a small number of muscles (Figures 4A,B). However, there were some differences in cluster composition, compared to the unobstructed condition as can be seen by examining the legend identifying the clusters in Figures 4A,B. These differences are illustrated in more detail in Figure 5 where the synergies identified during the swing phase in the unconstrained condition (thin ellipses) are compared to those obtained in the constrained condition (thick ellipses) for the lead (Figure 5A and trail (Figure 5B) conditions (see also Table 1).

**Table 1:** **Phase differences between the centroids of constrained and unconstrained clusters**.

**Constrained synergy**	**Unconstrained synergy**	**Difference phase**
**(A) LEAD CONDITION**		
1 (Bic)	1 (Bic)	0.0
2 (EDC, AcD, TrM, LtD)	2 (EDC)	0.059
2 (EDC, AcD, TrM, LtD)	3 (AcD, TrM, LtD)	0.014
3 (BrR, Prt, SpD)	4 (BrR, PrT, SpD, Br)	0.008
4 (Br)	4 (BrR, PrT, SpD, Br)	0.027
5 (Bic, ClB, ClT)	5 (Bic, ClB, ClT)	0.0
6 (LvS)	6 (LvS)	0.0
7 (ECR)	7 (ECR)	0.0
8 (Bic, EDC, PrT, TrM, ECR, LtD, SpD)	8 (Bic, EDC, PrT, TrM, ECR, LtD, SpD)	0.0
9 (SpD)	9 (SpD)	0.0
10 (ECU, PaL, SSp, TriL, Tri, AcD)	10 (ECU, PaL, SSp, TriL, Tri, AcD)	0.0
**(B) TRAIL CONDITION**		
1 (Bic, AcD)	1 (Bic, AcD)	0.0
2 (EDC, AcD, TrM, LtD)	2 (EDC, AcD)	0.057
2 (EDC, AcD, TrM, LtD)	3 (TrM, LtD)	0.046
3 (BrR, Prt, SpD, Tri)	4 (PrT, SpD, Tri, ECR, Br)	0.027
4 (Br)	5 (BrR, Br)	0.006
5 (Bic, ClB, ClT)	6 (ClB, ClT)	0.03
5 (Bic, ClB, ClT)	7 (Bic, LvS)	0.069
6 (LvS)	6 (Bic, LvS)	0.056
7 (ECR)	8 (ECR)	0.0
8 (Bic, EDC, PrT, TrM, ECR, LtD, SpD)	9 (Bic, EDC, PrT, TrM, ECR, LtD, SpD, AcD)	0.0
9 (SpD)	10 (SpD, ECU, PaL, SSp, TriL, Tri, AcD)	0.068
10 (ECU, PaL, SSp, TriL, Tri, AcD)	10 (SpD, ECU, PaL, SSp, TriL, Tri, AcD)	0.013

The tables show the differences in the phases of the centroids of the constrained and unconstrained clusters as illustrated in Figures 4, 5.

In the lead condition, there were only minor changes between constrained and unconstrained synergies as can be seen by the overlap of the ellipses representing the synergies obtained in the two conditions (Figure 5A) and by inspection of Table 1A. Indeed, 7 of the 10 clusters showed no changes at all in the two conditions (Table 1A). Among those clusters that were modified in the two conditions, the largest change was the division of cluster #2 (as defined in the unobstructed condition) into 2 clusters in the unconstrained condition. This was because of a slight phase advance of the EDC(1) burst with respect to the other three muscles in the cluster. Note, however, that even for this relatively large modification in phase, the change in centroid position of the EDC (0.059) was substantially less than the distance between the centroids of clusters #1 and #2 (0.13) and between clusters #2 and #3 (0.19) in the constrained condition. In addition, the Br was included in a cluster with the PrT and the SpD instead of being separate as in the unobstructed condition. This cluster in the unconstrained condition was only displaced by 0.008 from the location in the unconstrained condition (Table 1A). Despite these small changes in the unconstrained condition, it is evident that the basic elements of the synergies identified in the unobstructed condition are equally visible in the step over the obstacle.

Similar qualitative changes were seen in the trail condition as can be seen in Figure 5B. Again, the muscles comprising cluster #2 in the unobstructed condition were divided, this time into 2 groups of 2. Both clusters, however, remain well separate from the next cluster in the sequence. For example, the differences of the two unconstrained clusters (#2 and #3) from constrained cluster #2 was 0.057 and 0.046, respectively (Table 1B), while the distance between constrained clusters #2 and #3 was 0.17, three times as large. (The distance between cluster #1 and cluster #2 was 0.29). There were also changes in the other clusters, but in all cases, these involved the transfer of one muscle from one cluster into an adjacent cluster without change in the overall sequential nature of the activation of both individual muscles and clusters. In other words, in both the unobstructed condition and during the step over the obstacle, activation of e.g., LvS, follows activation of ClB and ClT, which follow activation of Br, which follows activation of PrT and SpD … etc.

Overall, the underlying principle of sequential activation of sparse synergies is well supported by the analysis (see Discussion). In particular, the analysis serves to illustrate that the synergies observed during the voluntary gait modifications are fully consistent with those observed during the unobstructed condition. More specifically, they serve to illustrate that the changes in the pattern of EMG activity that results in the modified limb trajectories required to step over the obstacles can be produced by changing the phase of activity of the synergies that underlie the EMG pattern during unobstructed locomotion.

To directly compare the phases of activity of the synergies in the lead and trail conditions with those identified during the unobstructed condition, we transformed the clusters obtained by using the constrained analysis into DCs, centered on the centroids of each cluster (Figures 6A–C) as described in the Methods (see also Krouchev et al., 2006; Drew et al., 2008a). These DCs, for each of the three conditions, are shown superimposed in Figure 6D, which allows the changes in phase of activation of each group of synergies to be synthesized in an economical manner. Changes in amplitude are ignored in this display but are addressed below. During the lead condition (red traces) the changes in phase are relatively minor apart from the phase delay in clusters 7 and 8, which are responsible for the wrist dorsiflexion and the preparation of the limb for landing. There is an increase in the duration of clusters #5 and #6, which include the Bic and the ClB (see Figure 2), and which are responsible for transporting the limb over the obstacle. In the trail condition, the phase advances observed in the activity of individual muscle bursts (Figure 3) have a substantial effect on the phase activity of a number of different synergies, and particularly those active prior to the onset of swing (i.e., Clusters #1–3). Note that treating cluster #2 as separate clusters as suggested by Figures 4A,B, 5 does not change the basic conclusion of a phase advance of muscle activity in this phase of the step cycle.

A One-Way Anova for phase of onset, phase of offset and peak phase (three different tests) as a function of locomotion condition showed significant differences for all combinations except 1 (phase of offset for synergy #9). Pair-wise comparisons of the three conditions likewise showed significant changes of at least one of the measured variables (onset, offset, peak) for all three comparisons (control-lead, control-trail, and lead-trail). In the control-lead comparisons, phase of onset was unchanged for four synergies while phase of offset was changed for all 10 synergies, reflecting the fact that most changes occur after swing onset in this condition. In the control-trail comparison, phase of offset was unchanged in three synergies. Overall these results emphasize that gait modifications require changes in the phase of activation of all synergies.

### Changes in amplitude of synergies during gait modifications (lead and trail conditions)

We measured the magnitude of the EMG activity of each burst of activity used in the analysis for the unobstructed, lead and trail conditions by integrating each 1 ms bin of activity between the measured onset and offset of the period of activity. These values were then divided by the duration of the burst (in ms) to give a value that represents the level of activity of the muscle burst in each condition, independent of any changes in duration of the muscle activity.

The results from this analysis are shown in Figure 7A, with each measured EMG burst being classified according to the synergy to which it is assigned. As expected from the data illustrated in Figure 3, muscles such as the Br, ClB and ECR(2) show a large increase in activity (up to 200%) during the lead condition while others, such as the PrT(1), and the TrM(1) show their largest changes (up to 400%) during the trail condition. In most cases, these changes were significantly different (asterisks) from control levels in both the control and lead conditions. Furthermore, in many cases, muscle amplitudes were significantly different between lead and control. Note that changes in the magnitude of activity cannot be displayed for muscle bursts that were inactive in the unobstructed situation.

In general, changes in magnitude of muscle bursts (relative to unobstructed locomotion) for clusters comprised of several muscles (e.g., clusters 2, 5, 8, and 10) were of the same sign and of similar magnitude. For example, in cluster #2 all of the muscles in the cluster showed a significant increase in activity during the trail condition. In clusters #3 and #5, each muscle shows a significant increase in the lead condition while in synergy #10, the magnitude of muscles in the lead condition is either unchanged or shows only a small increase. Only in cluster #8, does one muscle show a significant decrease in activity during the lead condition while the other five muscles either show an increase or are unchanged. A One-Way ANOVA for the changes in amplitude as a function of condition (control, lead, and trail) showed significant changes for all 10 synergies. Comparison between the pairs of conditions showed significant changes in at least seven synergies in all three conditions.

These results indicate that the voluntary gait modifications require changes in the magnitude of muscles in most synergies and that these changes in magnitude are different for the lead and trail conditions.

To provide an illustration of how control signals would need to change in both magnitude and phase in order to produce gait modifications, we used the average value for the EMG bursts in each cluster to scale the amplitude of the DCs that we illustrated in Figure 6. The results from this procedure are illustrated in Figure 7B which serves to illustrate that the gait modifications require coordinated changes in both amplitude and phase of the synergies. In the lead condition, the changes in phase or amplitude in the first two synergies (mainly responsible for paw lift) were small. Subsequently, there were changes primarily in amplitude in synergies 3–6 that are responsible for flexing the limb above the obstacle and transporting it forwards. Subsequently in synergies 7 and 8, responsible for wrist dorsiflexion and then placement of the paw on the substrate, there are increases in amplitude and a phase delay of synergy 7. Minimal changes are observed in the subsequent period of extensor muscle activity. In contrast, in the trail condition, there are both phase advances in the first 3 clusters and an increase in magnitude in clusters #2 and 3. There are minimal changes in phase or magnitude in clusters 4–6 although the slight decrease in duration of cluster #5 leads to a phase advance of the activity in clusters 7 and 8.

### Effect of obstacle dimensions

Changing the shape and size of an obstacle changes the limb trajectory as the cat steps over it (Drew, 1988). This implies that the relative magnitude, duration and timing of bursts of EMG activity in each synergy have to be precisely modulated to produce the appropriate limb trajectory. This is illustrated in Figure 8 with a different cat (MC8) for which data were available from a more limited number of muscles but for a variety of obstacles (Figure 8E).

Figure 8A illustrates EMG activity from representative muscles during control locomotion (black trace) and during steps over a thin high obstacle (Figure 8Eiii) in the lead (red trace) and trail condition (green trace). As in the example illustrated in Figure 3, there were characteristic changes in activity with phase advance of the TrM burst prior to swing onset in the trail condition, together with an increase in EMG amplitude of the ClB and phase delay of the burst of the EDC in the lead condition. Performing the cluster analysis on the EMG activity during unobstructed locomotion (Figure 8B) provides the same sequential pattern of activity that was obtained with our full database, despite the smaller number of muscles bursts that were available in this cat. However, because of the smaller number of EMG bursts in this database we identified only seven synergies.

The changes in phase and amplitude in the DCs constructed from these clusters are illustrated in Figure 8C for the lead condition and in Figure 8D for the trail condition. For all of the obstacles for which data were analysed these changes are similar in form to those obtained with our main database synthesized in Figure 7B. Anovas showed a significant effect of both condition and obstacle on the phase and magnitude for many of the synergy. In the lead condition, the effect of obstacle was significant for synergies #3, 6, and 7. In the trail condition, there was an effect of obstacle on all seven synergies.

In general, the change in phase and magnitude were smallest for the small high obstacle (green) and largest for the very high (mauve) and round (orange) obstacles. In cluster #3, in the lead condition, for example, there is a large increase in magnitude for the very high obstacle, presumably because of the need for shoulder retraction to raise the limb above the obstacle. In clusters #4 and 5, there are large changes in amplitude and duration for the 3 largest obstacles and relatively smaller ones for the small high obstacle. In cluster #6 there are large changes for the very high and round obstacles that require the paw to be raised above the obstacles and correspondingly large phase delays in cluster #7, especially for the wide obstacle. In the trail condition, changes in amplitude of cluster #3 are particular clear for the very high obstacle and to a lesser extent in the small high and the round obstacle. The changes in amplitude are least for the wide obstacle (which was also the least high). These changes are needed to retract the limb above the obstacle. The changes in duration are minimal except for the ECR (cluster #6). This latter is presumably because of the greater excursion required to bring the wrist into dorsiflexion in the trail condition (Lavoie et al., 1995).

## Discussion

We have argued in previous publications (Krouchev et al., 2006; Drew et al., 2008a) that small modifications in the activity of the sparse synergies defined by our analysis could provide a flexible manner of producing modifications in limb trajectory during walking. The results obtained in the current study support this premise by clearly demonstrating that small changes in the phase and magnitude of the synergies identified during unobstructed locomotion can produce the changes in limb trajectory required to step over obstacles of different sizes and shapes, both in the lead and the trail condition.

### Synergy definition and composition

Our definition of a synergy is a group of muscles that become active simultaneously and remain active for the same period of time. One of the results of this definition is that our synergies include only a limited number of muscles (hence sparse synergies) compared with those defined by mathematical decomposition that generally contain all muscles contained in the dataset (see below). Indeed, some of our synergies contain only a few, or in some cases, only one muscle (e.g., Cluster #7 in Figure 2). As we have argued previously (Krouchev et al., 2006), this is simply the result of recording a limited number of muscles and one would expect other wrist and digit extensors to be included in this synergy, if recorded. A correlate of using our definition of a synergy is that we define a relatively large number of synergies (10–11) compared to the 4–6 that are normally identified by the more commonly used decomposition methods (d'Avella et al., 2003; Tresch and Jarc, 2009). It is, however, interesting to note that synergies with similar compositions to those obtained using our methods can be obtained if the number of synergies defined by the linear decomposition methods is increased to 10 or 11 (Krouchev et al., 2006). Similarly, synergies comprised of a limited number of muscles can be obtained by other methods by including only those that show significant differences from lower amplitude weights (Hart and Giszter, 2004).

In this study we have taken the approach of defining our synergies on the basis of the muscle activity patterns generated during a single behavior, unobstructed locomotion, and then defining how these synergies must be modified to produce other behaviors. This differs from the more common approach in which synergies are defined on the basis of all behaviors under study (see e.g., d'Avella and Bizzi, 2005. In other words, most studies ask what parameters (weighting and phase) applied to all muscles in the dataset will result in all behaviors under study. In contrast, the question that we are asking is, how does one need to modify the magnitude and phase of the synergies obtained during unobstructed locomotion in order to obtain the patterns observed during voluntary gait modifications?

One of the results of defining synergies on the basis of a single behavior is the possibility that in addition to magnitude and phase, synergy composition may also be modified in other behaviors (see e.g., Ivanenko et al., 2005; Kargo and Giszter, 2000, see below). Indeed, we observed some changes in synergy composition in the unconstrained condition of our analysis (Figures 4A,B, 5). This raises the question of whether the voluntary gait modifications are really the result of modifying synergies that define unobstructed locomotion or whether there is a real need to modify synergy composition as suggested by Figures 4A,B. In some cases, such as the division of cluster #2 in the unobstructed condition into two clusters (#3 and #4) in the voluntary gait modifications, there is reason to think that this might indeed reflect a true need to modify the synergies. In most other cases, however, such as the merging of clusters 4 and 5, it is probable that this reflects the variability in the measurements of the onset and offset of EMG activity together with the modifications of the adjacency table because of the addition of bursts of activity not present in the unobstructed condition. Certainly in the absence of additional independent methods to define synergies this must remain an open question. However, what is clear is that the basic sequential activation of the muscles bursts and resultant synergies observed in the unobstructed condition is maintained in the voluntary gait modifications. For example, all 4 of the muscles comprising cluster #2 in the unobstructed condition are activated subsequent to the activation of the Bic(1) and prior to the activation of the Prt and SpD. Moreover, all 4 of these muscles show similar changes in magnitude during both the lead and the trail condition (Figure 7A). Lastly, as illustrated in Figure 6D, treating these muscles as 2 clusters does not alter the basic facts that the muscles active at this time are phase advanced compared to the unobstructed condition. Similar considerations hold for the other synergies. For example, whether the Br forms part of a synergy with the PrT (Figure 5A) or is in a separate synergy (Figures 2, 5B) does not change the fact that activity in both of these muscles occurs after the period of activity in the EDC and the TrM and before the period of activity in the ECR. Thus the basic concept of a sequential activation of a (relatively) large number of sparse synergies is fully supported by the data while the exact composition of each synergy must remain open to further investigation. Nonetheless, by maintaining the same number of synergies and the same composition, this approach has the advantage of allowing us to directly address some of the properties of the descending signals that would be required to modify gait by modifying the phase and magnitude of muscle synergies.

### Cortical control of synergies

Our results demonstrate the existence of a relatively large number of synergies, each of which is comprised of a small number of muscles (sparse synergies) and each of which is active during only a small part of the step cycle. These synergies are activated sequentially during the step cycle and show well defined modifications during the gait modifications.

In the lead condition, the major change is the increased duration and the phase delay of the synergies active at or after swing onset. These changes lead to the increased swing duration and the delayed onset of the activity of the muscles related to paw placement. There is also an increase in the magnitude of the synergy that leads to increased elevation over the obstacles. These changes in phase and magnitude are scaled to the size and the shape of the obstacle. Wider obstacles lead to progressively increased durations and longer phase delays. Higher obstacles lead to smaller changes in duration and smaller phase delays but increased amplitude in synergies related to limb flexion. In contrast, during the trail condition, the major changes are the phase-advance and increased amplitude of the synergies active before swing onset, particularly those active at paw lift and responsible for lifting the paw above the obstacle. The data therefore illustrate how differentially modifying the activity of different synergies can lead to adaptive changes in limb trajectory that allow for avoidance of obstacles covering a relatively wide range of sizes and shapes. There seems little doubt that the phase and magnitude of these synergies could be modified to produce an infinite range of limb trajectories that could be adapted to any specific obstacle that was encountered during locomotion.

**Figure 9:**
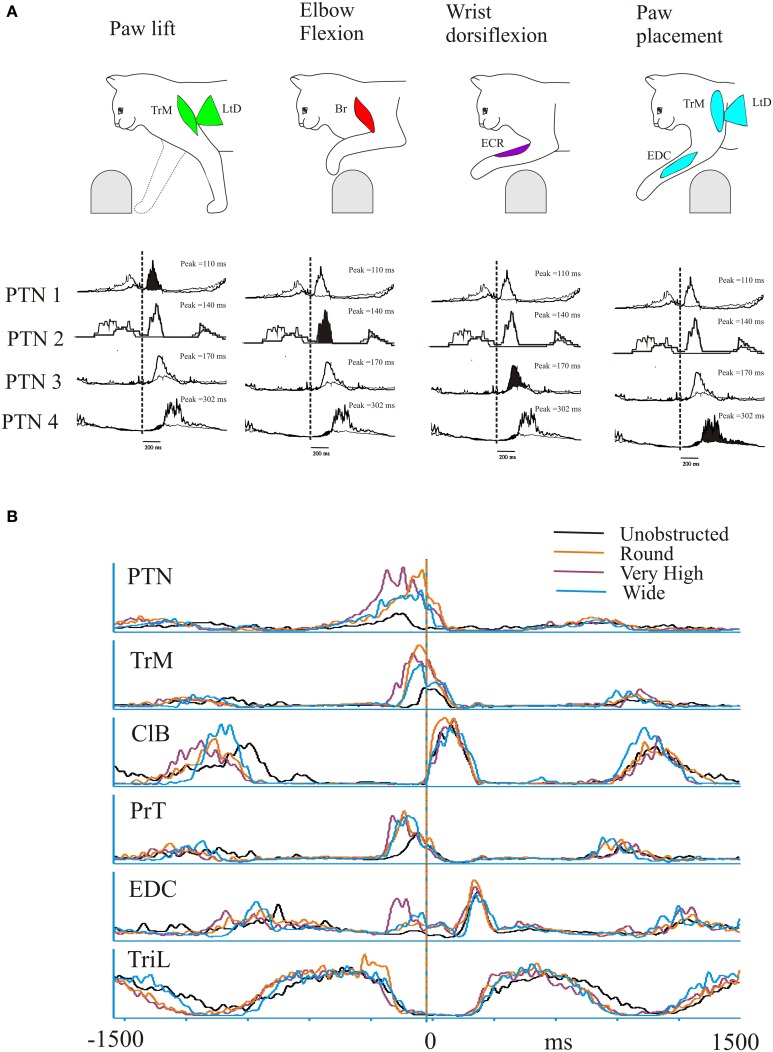
**Discharge activity of a pyramidal tract neurone (PTN) during the trail condition. (A)** (top) a representation of the sequential activation of selected muscles during a step over an obstacle. Below each figurine we illustrate the averaged activity of 4 PTNs, whose activity is synchronized to the onset of activity in the ClB (vertical dotted line). Each PTN is activated sequentially (filled histogram) as the cat steps over the obstacle. (Figure adapted from Drew et al., 2008b). **(B)** We illustrate activity of a PTN (different from those illustrated in **(A)** and five representative muscles during unobstructed locomotion (black trace) and during the trail condition during steps over the round (orange) very high (mauve) and wide (cyan) obstacle. Data are synchronized to the onset of activity in the ClB and are illustrated for 1500 ms before and after this event. Note the parallel changes in the magnitude and phase of the PTN and the TrM. The data for these illustrations is taken from a previous publication (Drew, 1993).

In the most parsimonious reasoning, one might expect that the activity of these synergies would be modified by control signals with similar characteristics. This is exactly what is seen in recordings from the cat motor cortex during locomotion (Drew, 1993; Drew et al., 1996, 2008a). During voluntary gait modifications, a large population of motor cortical cells (86% in Drew, 1993) show a modification in activity (increase or decrease) consisting of changes in magnitude, duration and/or the relative timing (phase of onset) of the discharge (Drew, 1993; Drew et al., 1996). Sub-populations of motor cortical cells recorded during locomotion, and particularly during gait modifications, are each activated during only a small part of the step cycle in a sequential pattern (Lavoie and Drew, 2002; Drew et al., 2008b). Examples of these activity patterns during the lead condition taken from the database used in a previous publication (Drew, 1993) are summarized in Figure 9A which serves to illustrate both the discrete, phasic nature of the activity patterns observed in different pyramidal tract neurones (PTNs) in the motor cortex and the sequential nature of this activation pattern. PTN1, for example, is active at the onset of the swing phase as the paw is lifted from the support surface at the onset of swing. This discharge occurs at the same time as the activity observed in muscles such as the TrM and the LtD. Subsequently, other PTNs become active coincident with the elbow flexion, characterized by activity in Br (PTN2); then with the wrist dorsiflexion, characterized by activity in the ECR (PTN3) and finally during paw placement, characterized by activity in muscles such as the EDC, TrM and the LtD (PTN4).

Particularly striking in our database (Drew, 1993) were cells whose discharge activity covaried with the first period of activity in the TrM (e.g., PTN1 in Figure 9A). As illustrated in Figure 9B these cells generally show a phase advance together with a major increase in the magnitude of their discharge in the trail condition, in the same manner as does the activity in synergy #3 (Figures 6, 7), containing the TrM. Importantly, discharge activity in these cells is scaled for different obstacles in the same way as the muscle synergy.

Overall, the data from our previous recording studies suggest that there are multiple sub-populations of PTNs, each of which regulates the activity of a specific sparse synergy. We suggest that there should be as many sub-populations of cortical neurones as there are synergies. However, we emphasize that some of the neurones that we recorded did not correlate with the activity of any of the muscles recorded. Such neurones might be related to controlling higher-level aspects of the movement or may even relate to more general kinematic variables (see Morrow et al., 2007).

The changes in phase and magnitude of cell discharge frequency of the overall population of task-related PTNs suffice to modify appropriately activity for those muscles in a synergy that are active in all three conditions (lead, control, and trail). A question arises, however, as to how muscles active only during the voluntary gait modifications are controlled. This is a particularly important consideration for those muscles that are strongly activated only in the trail condition, e.g., the Tri(1) burst. There are several possible ways in which this burst could be controlled. First, it is possible that cells that contribute to the production of activity in the extensor muscles during stance would discharge an additional burst of activity in the swing phase during the trail condition and contribute to activity both during swing and stance. However, an examination of the database of cells used in previous publications (Drew, 1993; Drew et al., 1996) showed little evidence for this pattern of activity. A second possibility is that there is a separate population of cells that discharge only, or primarily, in the trail condition and are responsible for the production of these additional bursts of activity. Again, there was little evidence for this in our population. A third possibility is that the discharge activity in the population of cells that influence activity in e.g., the TrM(1) is sub-threshold to produce activity in e.g., the Tri(1) during the unobstructed and lead conditions. The large increase in activity observed in these cells during trail, however, would be sufficient to produce a (non-linear) increase in the excitation of this muscle. Such a non-linear effect might be facilitated by a change in afferent input produced by the modified movement. It is interesting to note, for example, that low intensity stimulation of peripheral afferents during the swing phase of locomotion evokes strong responses in the Tri, despite the lack of any natural activity in the motoneurones at this time (Drew and Rossignol, 1987).

**Figure 10:**
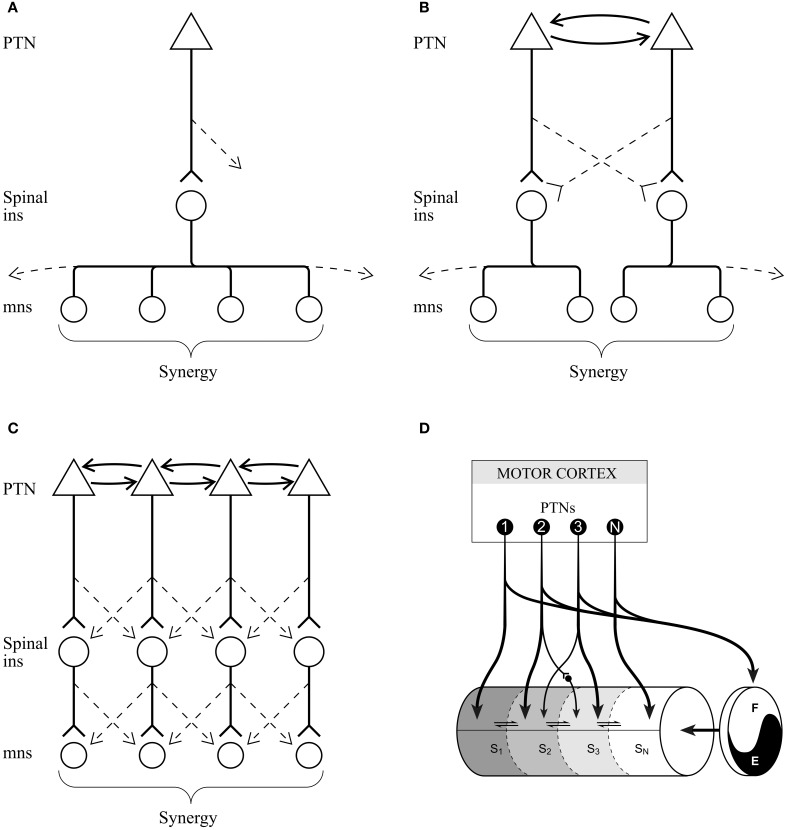
**Schematics to illustrate possible corticospinal connections. (A)** a single subpopulation of PTNs connects with spinal interneurons (ins) that project to the motoneurones (mns) of all muscles in a synergy. **(B)** Two subpopulations of PTNs, linked by intracortical connections, and discharging simultaneously during locomotion connect with different populations of spinal interneurons, each of which innervate only part of the total muscle synergy. **(C)** Same principal as **(B)** but providing the possibility of more fractionation. Dotted lines in **(A–C)** indicate weaker connections to interneurons and motoneurones that are not part of the synergy directly modified by the motor cortical signal. **(D)** conceptual model illustrating the interaction of sub-populations of PTNs with spinal modules comprising the CPG. Each module will activate the motoneurones of a given synergy. Note that in this illustration we have separated the rhythm generator from the pattern generator elements as in the model by Rybak et al. (2006) and as supported by the results of our microstimulation studies (Rho et al., 1999). **(D)** is modified from Drew (1991a); Drew et al. (2008b). Abbreviations: E, extensor part of rhythm generator; F, flexor part of rhythm generator; in, interneurone; mn, motoneurone; PTN, pyramidal tract neurone; Sn, synergy #n.

In addition to issues of the characteristics of the control signal, one also has to consider the anatomical bases for the proposed control system. If a given synergy is controlled as a unit by activity in a small homogeneous subpopulation of PTNs, then one would expect one or both of the following conditions to be met: (1) given that there are no monosynaptic connections from the motor cortex to motoneurones in the cat (Illert et al., 1976), PTNs should project to interneurons in the spinal cord that selectively activate the muscles identified as belonging to a single synergy (Figure 10A) and/or (2) cells in the motor cortex with more restricted projections to a subset of the muscles identified as belonging to a single synergy are connected by intra-cortical connections (Figures 10B,C). General evidence for both of these propositions exists. The anatomical studies of Alstermark (Tantisira et al., 1996) and the electrophysiological studies of Lundberg et al. (1987) emphasize that interneurons within the spinal cord branch to activate groups of proximal and distal muscles. In addition, Shinoda has very elegantly shown that individual corticospinal axons branch to multiple levels of the cervical spinal cord (Shinoda et al., 1976, 1986; Futami et al., 1979). Together, this provides two mechanisms by which small populations of cortical neurones could activate muscle synergies. Interestingly, Hart and Giszter (2010) have used spike triggered averaging (STA) to demonstrate that the muscles activated by a given interneurone in the frog spinal cord correlate strongly with the weighting matrix of individual motor primitives, or synergies. Furthermore, in the primate, STA demonstrates that individual corticomotoneuronal neurones in the motor cortex can influence the activity of multiple muscles including those acting at proximal and distal joints (McKiernan et al., 1998; Griffin et al., 2008). Similarly, microstimulation of the motor cortex, particularly during locomotion (Armstrong and Drew, 1985) simultaneously activates both proximal and distal muscles. At the cortical level, there are abundant references to show the richness of the cortico-cortical connections between both adjacent and more distant regions of cortex (Huntley and Jones, 1991; Schneider et al., 2002; Capaday et al., 2009, 2011; Smith and Fetz, 2009). In addition, the motor cortex receives abundant input from premotor cortex and from the posterior parietal cortex (Ghosh, 1997; Andujar and Drew, 2007), which might also serve to coordinate activity between different subpopulations of PTNs.

Missing from the experimental data in the mammal, however, is the information as to whether the muscles linked anatomically or functionally in the studies mentioned above correspond to the synergies identified by our, or other, studies. In addition, there is no evidence of a direct link between the discharge pattern of individual cells and the muscles of the synergy that is activated or influenced by its discharge.

### Comparison with other models of modularity

Our model shares the same basic concept proposed by Bizzi (see Introduction for references), namely that a complex series of behaviors can be produced by the differential combination of a finite number of synergies. This base concept has been tested and confirmed in a large number of behaviors as listed in the Introduction. However, the majority of these models, whether based on synchronous or time-varying synergies (d'Avella et al., 2003), propose a small number of synergies, typically 4–6, although sometimes fewer, to control a wide range of complex movements. Moreover, the control signals, represented by the waveforms resulting from the mathematical decomposition, especially those obtained using principal component analysis (PCA), are generally quite broad and occupy a large proportion of the step cycle (e.g., Lacquaniti et al., 2012) or the movement phase (e.g., Overduin et al., 2008). These control signals activate muscles according to a weighting matrix. As such, all muscles are activated to varying amounts by all control signals and the movement produced is the sum result of the muscle activation although generally a small number of muscles has relatively large weights in each synergy (see e.g., Kargo and Nitz, 2003; Hart and Giszter, 2004). Moreover, the calculation of the synergies is generally performed by including as wide a range of movements as possible to ensure that the resultant synergies are capable of reproducing a large behavioral repertoire. It is generally considered that these signals are then transformed at the level of the spinal cord to produce the appropriate periods of muscle activity (see e.g., Ting, 2007 for a schema for postural control). Some support for this view of motor control comes from recent experiments by Overduin et al. (2012) who showed that the EMG responses evoked by long trains of stimulation (150–500 ms) at distributed points in the primate motor cortex could be reduced to three synergies. Moreover, the synergies evoked by microstimulation corresponded to the synergies defined by EMG activity during reach and grasp movements.

In contrast, our approach and resulting conceptual model differs in two important considerations from those of the more commonly used methods. The first is that we use a more classical method of defining a synergy (see above) and place no limit on the number of synergies that can be defined. During locomotion, this results in the identification of 10 or 11 synergies rather than the 4–6 more commonly used. Moreover, the large number of synergies and the fact that a single muscle burst cannot be included in more than one synergy results in the sparse synergies defined in this manuscript and in Krouchev et al. (2006). These synergies occupy only a small part of the step cycle and are compatible with a control mechanism by which each synergy is modulated by a specific sub population of cortical neurones as defined in the previous section. The second difference is that we define our synergies during only a single behavior, locomotion. This is based on the premise that the neural circuits in the spinal cord primarily evolved to be optimal for producing the basic pattern of locomotion. As such, we propose that the motor cortex (and other descending pathways) act via these circuits, adapting their activity to the behavior as required. In some cases, as demonstrated here for voluntary gait modifications, modifying the phase and magnitude of these synergies is sufficient to produce changes in behavior. In other behaviors, however, the challenge might well be to modify these synergies by changing both the number and the composition of these synergies (see also Kargo and Giszter, 2000, 2008). This contrasts with the idea of a limited number of immutable synergies that can be used to produce a wide range of behaviors (d'Avella and Bizzi, 2005; Overduin et al., 2008).

Our results can also be compared to other studies and in other tasks that have specifically studied the contribution of motor cortical discharge to the control of synergies. Kargo and Nitz (2003), for example, also showed motor cortical cells that were sequentially activated in rats trained to reach. They used independent component analysis (ICA) to identify a number of synergies active throughout a reach. Cross-correlation analyses showed that a majority of task-related motor cortical cells showed a significant correlation with only a single independent component (IC). When cross-correlated with the activity of individual muscles, neurones were significantly correlated with many, although not all of the muscles included in a synergy. Miller (Holdefer and Miller, 2002; Morrow et al., 2009) also suggested the existence of a number of muscle synergies during a reaching task, with each synergy controlled by separate populations of motor cortical neurones. These synergies were relatively stable across task (Morrow et al., 2009). In our own work (Yakovenko et al., 2011), using similar methods to those in this manuscript, we have also shown sparse synergies during reaching movements that are similar to those observed during locomotion and we have suggested that PTNs in the motor cortex control the limb trajectory during reaching in the same manner that we propose for voluntary gait modifications.

The model that we propose is clearly most similar to the original modular concepts proposed by Grillner (1981). As illustrated in Grillner and Wallen (1985), we propose that descending pathways act via modules in the spinal cord to modify locomotion. We further propose that the detailed motor commands observed in sub-populations of motor cortical neurones (Drew, 1993; Drew et al., 1996, 2004, 2008b) provide a means for exerting specific control over muscle activity while ensuring that the resulting gait modifications are fully integrated into the base locomotor rhythm (Drew, 1991a; Figure 10D). However, it should also be noted that in the majority of these experiments, again including our own, the synergies that are identified are multi-articular and not confined simply to a single joint as in the original model of Grillner (1981).

It could be argued that the synergies that we are defining in the intact cat emerge from the concerted activity of a unit CPG of the type produced by Griller, moulded by descending and peripheral afferents to produce our more fractionated, multi-joint modules. Indeed, a recent study by Markin et al. (2012), using methods similar to those described here, found some differences in the synergies identified in the hindlimb of intact cats and those identified during fictive locomotion in decerebrate cats. These differences were primarily observed in muscles acting around two joints suggesting that the expression of the final pattern of activity in such muscles is modified by peripheral input. In this respect, it is important to realize that the patterns of activity observed in spinal animals are strongly modulated by peripheral input (Pearson and Rossignol, 1991; Rossignol et al., 1993; Lemay and Grill, 2004; Saltiel and Rossignol, 2004a,b; Cheung et al., 2005).

### Synergies as a unit of control of general motor activity

Even in cats a large number of forelimb movements do not involve the simple sagittal pattern of intralimb coordination that we have so far examined and in primates, and especially in humans, the patterns of limb movement become more and more flexible. This is especially true when we consider grasping movements that involve both arm movement and control of the hand. Indeed, the extent to which control of hand movements may be explained by synergies is quite controversial (Brochier et al., 2004; Theverapperuma et al., 2006; Kutch et al., 2008; Overduin et al., 2008). How then does the concept of a control of movement by synergies, at least those of the type that we propose, lend itself to a flexible control of limb movement throughout a wide behavioral range?

The answer to this question lies to some extent in whether one considers muscle synergies as a concept that simplifies motor control, as is generally assumed (see Tresch and Jarc, 2009) or one that is the result of an evolution in which more recently developed descending pathways must act through spinal circuits that have been conserved (see Krouchev et al., 2006; Giszter et al., 2007; Giszter and Hart, 2013). As mentioned above, we favor the second possibility. We suggest that with the parallel evolution of the nervous system and the musculoskeletal system, spinal circuits became adapted to provide more flexible and agile locomotor limb movements, culminating in reaching and ultimately, in primates, reaching and grasping movements (Georgopoulos and Grillner, 1989). In this view, as movements became more complex, the challenge for the nervous system was to produce flexible movements that are independent of the more stereotypical arm movements observed during locomotion. Indeed, from an evolutionary viewpoint one might speculate the development of a hierarchical control system that evolved together with the increasing flexibility required in the control of movement. Such a hierarchy is frequently discussed in the locomotor literature (Rossignol, 1996) but less frequently with respect to voluntary movements (see Ting, 2007; Roh et al., 2011).

During locomotion, it is well established that circuits in the spinal cord generate a rhythm that contains details about the pattern of muscle activity (see Introduction). These spinal circuits are then subject to modification by brainstem and cortical inputs. The brainstem pathways are suggested to regulate the level of muscle activity and posture, for example, during walking uphill (Orlovsky, 1972; Drew et al., 2004). This general control of muscle activity is facilitated by the fact that the axons of neurones in the brainstem pathways, the reticulo- and vestibulo-spinal tracts, have diffuse termination patterns that influence muscle activity around multiple joints, and in multiple limbs (Matsuyama et al., 1988; Drew and Rossignol, 1990a,b). Moreover, the signals recorded from reticulospinal neurones during unobstructed locomotion (Drew et al., 1986; Matsuyama and Drew, 2000) and during voluntary gait modifications (Prentice and Drew, 2001) do not show the same level of fractionation as signals from the motor cortex. Indeed, many cells in the reticulospinal system show broad patterns of modulation that are more compatible with the waveforms identified by many of the decomposition studies than those observed in the motor cortex. Moreover, rather than specifying the exact patterns of motor activity that need to be produced, we have suggested that the signals from these pathways are integrated with the rhythmical signals in the spinal cord to modify the production of many of the more stereotypical patterns of behavioral activity, including locomotion (Drew et al., 2004). In some respects, therefore, the activity and connectivity of brainstem pathways resembles that postulated for synergies in general, and especially those implicating the brainstem (Roh et al., 2011). Nonetheless, it should be emphasized that there is little evidence for a limited subset of discharge patterns among reticulospinal cells during locomotion as might be expected if they represented a small range of synergies. Moreover, it should also be noted that synergy studies generally concentrate on the activity within a single limb whereas many neurones in the reticulospinal pathways appear to be involved in coordinating activity in two, or more, limbs.

At a higher level of the hierarchy, the motor cortex (and the red nucleus) provides a more specific level of control of locomotion. During unobstructed locomotion, the contribution is likely to be one of step-by-step regulation of the step cycle superimposed on the control exerted at lower levels of the nervous system. A contribution at this level is supported by the fact that motor cortical cells, including PTNs are modulated during unobstructed locomotion [reviewed in Drew et al. (1996)] and that microstimulation of the motor cortex can modify both the pattern and the timing of the step cycle (Armstrong and Drew, 1985; Rho et al., 1999). The major contribution of the motor cortex, however, is in the control of precise locomotor movements on the basis of visual information (Drew, 1988, 1993; Beloozerova and Sirota, 1993). As developed in this manuscript, we propose that the motor cortex produces gait modifications by altering the timing, duration and relative timing of muscle synergies. As suggested above, these signals are likely to be mediated by the same neuronal circuits responsible for the generation of the locomotor rhythm and the pattern of locomotion. This allows a specific control over the level of activity in the different synergies, thus adapting activity to the specific requirements of the task while still integrating this into the step cycle (Figure 10D). In our model, control of voluntary gait modifications does not require the production of new synergies but rather modification of the synergies already present in unobstructed locomotion. This should be compared with the results of Ivanenko et al. (2005) in which stepping over an obstacle by human subjects required the addition of a new principal component that was responsible for explaining >20% of the overall variance in the EMG patterns.

At the highest level there is a need to control movements that are non-stereotypic. This might involve breaking apart synergies or the addition of new synergies. For example, during corrective manoeuvres, Kargo and Giszter (2000) have shown there is a need to modify synergy composition to correct for perturbations. In other conditions, for example when making relatively simple movements, there might be a need to activate 1 or 2 sparse synergies independently of others. Other movements may require separation of synergies, for example, uncoupling activity in wrist and elbow muscles. This may be achieved by activating only a part of the population of PTNs contributing to muscle activity in a given synergy (e.g., Figure 10B). Additionally, it might require inhibiting some of the motoneurones that would normally be activated by a given sub-population of PTNs. Indeed, it has been suggested that the development of the motor cortex and, in primates, the corticomotoneuronal system, provides the ability to generate more fractionated movements, in part by inhibiting some synergies and activating others (Lemon and Griffiths, 2005; Drew et al., 2008a). In other movements, particularly those involving the fingers, the number of patterns of motor cortical recruitment makes it difficult to argue that movement is controlled by synergies. Poliakov and Schieber (1999), for example, failed to find any evidence of broad groups of motor cortical neurones controlling finger movements and instead reported a highly diverse pattern of activity suggesting control by a distributed network.

### Summary

The data illustrated in this manuscript are compatible with a view that motor cortical cells contribute to the control of locomotion by modulating the activity of a limited number of sparse synergies. We suggest that a relatively simple control system can serve to control limb trajectory under a wide range of situations simply by modifying the phase and magnitude of the period of activity in a limited number of functionally segregated populations of PTNs. In addition, Yakovenko et al. (2011) showed that similar synergies are observed during reach as during locomotion. Indeed, the sequential organization of sparse synergies in unobstructed locomotion, stepping over obstacles and even reaching, is strongly suggestive of a relationship to prime mover muscles and their agonists. These relationships, in turn, are activated subject to biomechanical (kinematic, dynamic) constraints and to existing neural circuits provided by subcortical and spinal neural pattern- and rhythm- generating circuitry.

### Conflict of interest statement

The authors declare that the research was conducted in the absence of any commercial or financial relationships that could be construed as a potential conflict of interest.
